# *Toxoplasma gondii* suppresses proliferation and migration of breast cancer cells by regulating their transcriptome

**DOI:** 10.1186/s12935-024-03333-1

**Published:** 2024-04-23

**Authors:** Hengming Ye, Xiaotao Zhou, Bike Zhu, Tiantian Xiong, Weile Huang, Feng He, Hui Li, Lihua Chen, Luying Tang, Zefang Ren

**Affiliations:** 1 The School of Public Health, Sun Yat-Sen University, 74 Zhongshan 2nd Rd, Guangzhou, 510080 Guangzhou China; 2 Public Health Service Center of Bao’an District, Shenzhen, 518102 China; 3 Shenzhen Hospital of Guangzhou University of Chinese Medicine (Futian), Shenzhen, 518034 China; 4 The Third Affiliated Hospital, Sun Yat-Sen University, Guangzhou, 510630 China

**Keywords:** *Toxoplasma gondii*, Breast cancer, Proliferation, Migration, Transcriptome

## Abstract

**Background:**

Breast cancer is the most common cancer in women worldwide. *Toxoplasma gondii* (*T. gondii*) has shown anticancer activity in breast cancer mouse models, and exerted beneficial effect on the survival of breast cancer patients, but the mechanism was unclear.

**Methods:**

The effect of tachyzoites of *T. gondii* (RH and ME49 strains) on human breast cancer cells (MCF-7 and MDA-MB-231 cells) proliferation and migration was assessed using cell growth curve and wound healing assays. Dual RNA-seq was performed for *T. gondii-*infected and non-infected cells to determine the differentially expressed genes (DEGs). Gene Ontology (GO), Kyoto Encyclopedia of Genes and Genomes (KEGG), and Protein–Protein Interaction Networks analysis (PPI) were performed to explore the related signaling pathway and hub genes. Hub genes were validated using the Kaplan–Meier plotter database, and Pathogen Host Interaction (PHI-base) database. The results were verified by qRT-PCR.

**Results:**

The tachyzoites of *T. gondii* decreased the expression of *Ki67* and increased the expression of *E-cadherin*, resulting in suppressing the proliferation and migration of infected human breast cancer cells. The inhibitory effect of *T. gondii* on breast cancer cells showed a significant dose–response relationship. Compared with the control group, 2321 genes were transcriptionally regulated in MCF-7 cells infected with *T. gondii*, while 169 genes were transcriptionally regulated in infected MDA-MB-231 cells. Among these genes, 698 genes in infected MCF-7 cells and 67 genes in infected MDA-MB-231 cells were validated by the publicly available database. GO and KEGG analyses suggested that several pathways were involved in anticancer function of *T. gondii*, such as ribosome, interleukin-17 signaling, coronavirus disease pathway, and breast cancer pathway. *BRCA1, MYC* and *IL-6* were identified as the top three hub genes in infected-breast cancer cells based on the connectivity of PPI analysis. In addition, after interacting with breast cancer cells, the expression of *ROP16* and *ROP18* in *T. gondii* increased, while the expression of *crt, TgIST, GRA15, GRA24 and MIC13* decreased.

**Conclusions:**

*T. gondii* transcriptionally regulates several signaling pathways by altering the hub genes such as *BRCA1, MYC* and *IL-6*, which can inhibit the breast tumor growth and migration, hinting at a potential therapeutic strategy.

**Supplementary Information:**

The online version contains supplementary material available at 10.1186/s12935-024-03333-1.

## Background

*Toxoplasma gondii* (*T. gondii*) is an obligate intracellular parasite belonging to the phylum *Apicomplexa*. Its main forms include oocysts, tachyzoites, cysts and bradyzoites [1]. *T. gondii* is predominately divided into three clonal lineages designated type I (highly virulent), II (avirulent) and III (avirulent) [2, 3]. This parasite has infected approximately one-third of the world's population through digestive tract transmission, blood transmission and congenital transmission [1, 4–6]. Generally, *T. gondii* infection is asymptomatic or self-limiting in immunocompetent population, but it can cause severe toxoplasmosis in immunocompromised hosts and severe birth defect in newborns [7, 8].

However, our recent study found that anti-*T. gondii* IgG was associated with a better survival of breast cancer patients, especially in women with high interleukin-17 (IL-17) or IL-9 levels [9]. Mountains of animal experiments have found that *T. gondii* can efficiently inhibit the growth and metastasis of several types of cancer such as ovarian cancer [10], pancreatic cancer [11] and breast cancer [12]. For example, after injecting with artificial attenuated *T. gondii* in the mice inoculated with 4T1 cells (murine triple-negative breast cancer cells), tumor growth and metastasis was suppressed by increasing the secretion of interleukin-12 (IL-12) and interferon-γ (IFN- γ), which may inhibit the angiogenesis and induce infiltrating T cells in tumor microenvironment [12]. In addition, some studies have found that *T. gondii* was capable of maturing the dendritic cells, which subsequently activate CD8^+^T cells to kill tumor cells [10].

Recently, a study compared the difference of transcriptome between *T. gondii*-infected mice and non-infected ones using RNA-seq. This study found that the expression of several genes related to breast cancer signaling pathway was dysregulated in infected group, such as *BRCA2* (up-regulated) and *CCND1* (down-regulated) [13]. *BRCA2* has been recognized as a tumor suppressor gene [14], while CCND1 is a possible oncogenic gene. Thus, this study suggests that *T. gondii* may reduce the risk of breast cancer by regulating transcriptomic expression. However, it remains unclear how *T. gondii* regulates the signaling pathway of breast cancer.

Accordingly, this study assessed the effect of tachyzoites of *T. gondii* on proliferation and migration of human breast cancer cells using cell growth curve and wound healing assays. Dual RNA-seq was further applied to analyze the transcriptomic changes of breast cancer cells and *T. gondii* after their interaction.

## Materials and methods

### Cell culture

Human breast cancer cells MCF-7 cell and MDA-MB-231 cell were kindly provided by Stem Cell Bank, Chinese Academy of Sciences (https://www.cellbank.org.cn/). Human foreskin fibroblast (HFF) cell was donated by Professor Hongjuan Peng from the School of Public Health, Southern Medical University [12]. MCF-7 cell and HFF cell were grown in RPMI1640 (cat.no. C11875500BT; Gibco, USA) with 10% fetal bovine serum (FBS; cat.no. SV30160; Hyclone, USA), 100 U/mL penicillin–streptomycin (cat.no. 15140122; Gibco, USA). MDA-MB-231 cell and HFF cell were grown in DMEM (cat.no. C11995500BT; Gibco, USA) with 10% FBS. All cells were cultured at 37℃ in a humidified CO_2_ (5%) atmosphere.

### Parasite culture

*T. gondii* RH strain and ME49 tachyzoites were maintained in HFF monolayers cultured in DMEM supplemented with 10% FBS (v/v). Freshly egressed parasites purified by passage through a 3-μm polycarbonate filter (Whatman, Chicago, USA) were used in all experiments [12].

### Cell infection and cell proliferation assay

MCF-7 and MDA-MB-231 cells were plated into 24-well plates (1 × 10^4^ cells/well). Then, the cells were infected with *T. gondii* RH strain at various concentrations (multiplication of infection, MOI = 1, 2, 4, 8 and16) for 7 days. To evaluate the impact of avirulent *T. gondii* strain on breast cancer cells, *T. gondii* ME49 strain was used to infect the breast cancer cells at various concentrations (MOI = 2, 4, 8, 16 and 32) for 7 days. All 24-well plates were maintained at 37℃ in a humidified CO_2_ (5%) atmosphere. The cell numbers were subsequently counted each day using the cell counting chamber. The presence of *T. gondii* was observed by Giemsa staining.

### Wound healing assay

The migration ability of the indicated cell was evaluated by the wound healing assay. Breast cancer cells were seeded in 6-well plates (2 × 10^6^ cells/well). When the cells reached 90–100% confluence in culture plate wells, linear scratch wounds were made in the cell monolayers with a 200-μL pipette tip. The cells in plate well were washed three times with warm phosphate buffered saline (PBS; cat.no. 10010023; Gibco, USA) to remove cellular debris. The cells were then treated with *T. gondii* (RH strain, MOI = 4; ME49 strain, MOI = 16) for 24 h. The images were acquired using a microscope (Nikon Eclipse TS-E; Nikon Instruments Inc., Melville, NY, USA) at 0, 6, 12, and 24 h after infection. The area of each scratch wound was determined by using ImageJ software.

### Isolation of RNA and quantitative real-time PCR

Total RNA was extracted using Trizol reagent kit (Invitrogen, Carlsbad, CA, USA) according to the manufacturer’s protocol. RNA quality was assessed on NanoDrop 2000 (Thermo Fisher Scientific, MA, USA) and checked using RNase free 1.5% agarose gel electrophoresis. Subsequently, the RNA was transcribed to first strand cDNA by the HiScript III 1st Strand cDNA Synthesis Kit (cat.no. R323‐02; Vazyme, Nanjing, China). For mRNA amplification, the validated primers obtained from PrimerBank (https://pga.mgh.harvard.edu/primerbank/) [15] were listed in Additional file 3: Table S1. PowerUp™ SYBR™ Green Master Mix (cat.no. A25742, Applied biosysytems, USA) was used to perform qRT-PCR (quantitative real-time PCR, qRT-PCR) on a real-time RCR cycler (QuantStudio 6, Thermo Fisher Scientific, USA). The amplification reaction reactions were performed using the following conditions: 50 ℃ for 2 min, 95 ℃ for 2 min followed by 40 cycles of 95 ℃ for 15 s, 60 ℃ for 1 min. Melting curve analysis was conducted using the following conditions: 95 ℃ for 15 s, 60 ℃ for 1 min, and 95 ℃ for 15 s. The expression levels of target genes were normalized to the internal control gene *β-actin* value*.* The relative expression of the genes was calculated by the 2^−ΔΔCT^ method [16].

### RNA-seq and bioinformatic analysis

mRNA libraries were constructed by using NEBNext Ultra RNA Library Prep Kit (NEB#7530, Illumina, New England Biolabs, MA, USA). Each library was sequenced using Illumina NovaSeq 6000 (Illumina, New England Biolabs, MA, USA). Adaptor sequences and low-quality reads were removed using fastp (v.0.18.0) [17]. Ribosome RNA (rRNA) was removed using short reads alignment tool Bowtie2 (v.2.2.8) [18]. Clean datasets were aligned with HISAT2 (v.2.2.1) [19] against three publicly available reference transcriptome: the human transcriptome from Ensembl database (Release-106, http://asia.ensembl.org/Homo_sapiens) and two *T. gondii* transcriptome from ToxoDB database (TgondiiRH88; TgondiiME49; version ToxoDB-59) [20]. For each transcription region, a FPKM [21] (fragment per kilobase of transcript per million mapped reads) value was calculated to quantify its expression abundance using RSEM software [22]. Differentially expressed genes (DEG) were detected by DESeq2 [23] using unpaired *t*-test with Benjamini–Hochberg correction, and the filter criteria was set as fold changes ≥ 1.5 and adjusted *p*-value < 0.05. The raw sequence data reported in this paper have been deposited in the Genome Sequence Archive [24] in National Genomics Data Center [25], China National Center for Bioinformation / Beijing Institute of Genomics, Chinese Academy of Sciences (GSA-Human: HRA004430; GSA: GRA010759) that are publicly accessible at https://ngdc.cncb.ac.cn/gsub. Subsequently, the enrichment analysis of Gene Ontology (GO) and KEGG pathways was performed based on these DEGs by using software GSEA [26] and the R package “ClusterProfiler” [27].

### Identification of hub genes regulated by T. gondii

After obtaining the DEGs between *T. gondii* infected and uninfected breast cancer cells, we integrated the DEGs into two groups of target genes: up-regulated group and down-regulated group. Then, we validated these target genes found in cell experiments as above by associating with tumor progression in a breast cancer cohort which was publicly available in the database of Kaplan–Meier plotter (KM plotter, https://kmplot.com/analysis/). This database contained the RNA-seq data and survival information of 2976 breast cancer patients from Gene Expression Omnibus (GEO) and European genome-phenome archive (EGA) [28]. Expression level of each gene was treated as a binary variable in the survival model, and the optimal cutoff was auto-selected by the KM plotter. Considering the regulating direction, we further screened out the validated genes from the overlapped set between the genes up-regulated by *T. gondii* and the significant ones associated with decreased risk of death, and vice versa. The STRING online database (https://string-db.org/) was utilized to construct the protein–protein interaction (PPI) network of the validated genes. Software Cytoscape (v.3.9.1) was used to identify the hub genes based on the degree of connectivity. Finally, for host-parasite interaction analysis, we retrieved human and their relevant *T. gondii* interactions from Pathogen Host Interaction (PHI, http://www.phi-base.org/index.jsp) database.

### Statistical analysis

Unpaired two tailed Student’s *t*-test or one-way ANOVA was performed to compare the gene expression levels between control and experimental groups. Unpaired two tailed Student’s *t*-test was also used to analyze the difference of wound healing rate between control and experimental groups. All analyses and visualization were performed using R version 4.1.2 and GraphPad Prism 8 (GraphPad Software Inc., CA, USA), and a two-sided *P* value below 0.05 was considered as statistical significance.

## Results

### *T. gondii* inhibits the proliferation of breast cancer cells

**Fig. 1:**
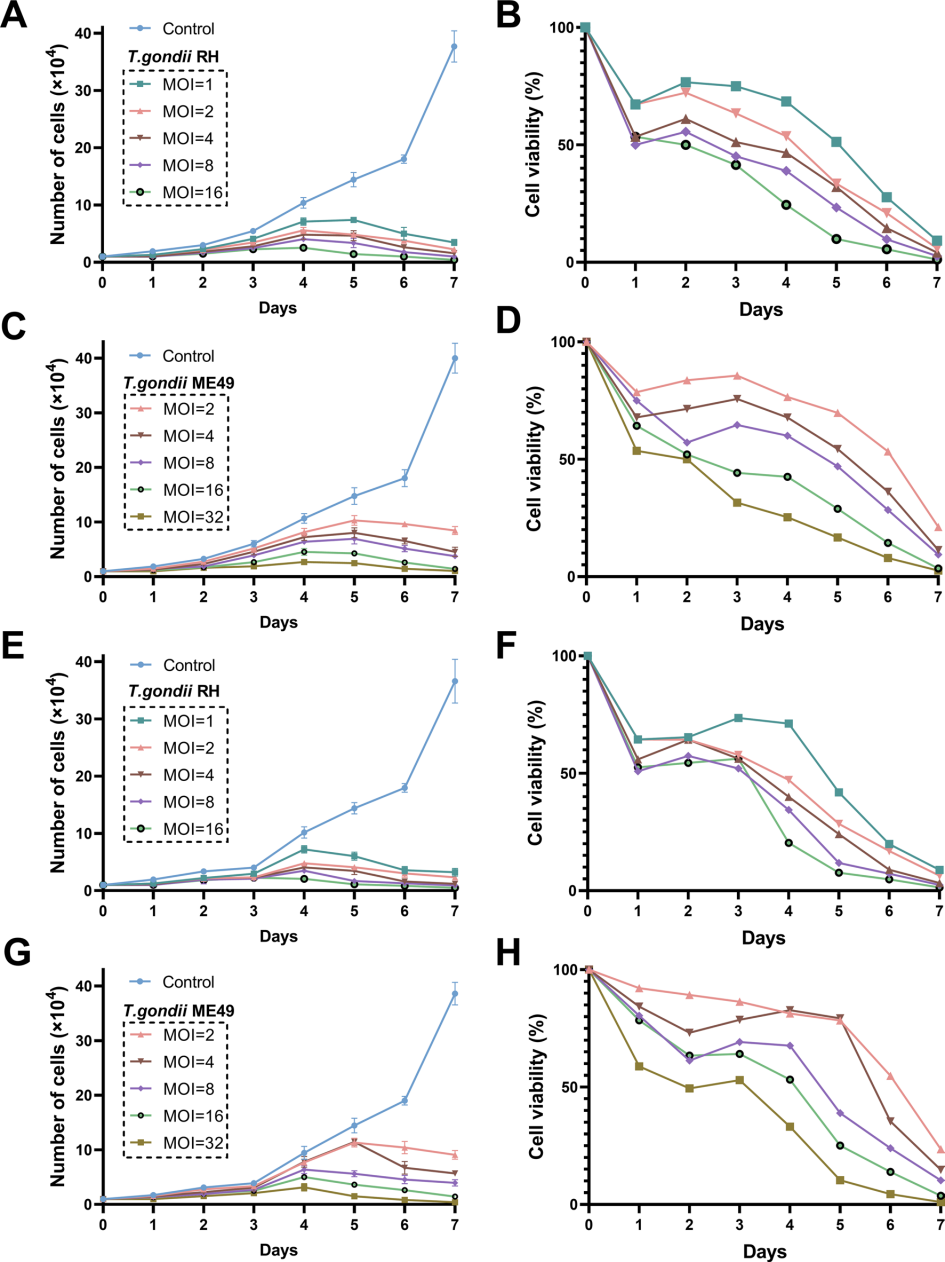
Breast cancer cells growth curve and cell survival curve after infection with *T. gondii*. **A** Cell growth curves of MCF-7 cells infected with *T. gondii* RH strains at various concentrations. **B** Cell survival curves of MCF-7 cells infected with *T. gondii* RH strains at various concentrations. **C** Cell growth curves of MCF-7 cells infected with *T. gondii* ME49 strains at various concentrations. **D** Cell survival curves of MCF-7 cells infected with *T. gondii* ME49 strains at various concentrations. **E** Cell growth curves of MDA-MB-231 cells infected with *T. gondii* RH strains at various concentrations. **F** Cell survival curves of MDA-MB-231 cells infected with *T. gondii* RH strains at various concentrations. **G** Cell growth curves of MDA-MB-231 cells infected with *T. gondii* ME49 strains at various concentrations. **H** Cell survival curves of MDA-MB-231 cells infected with *T. gondii* ME49 strains at various concentrations. MOI: multiplicity of infection. Cell viability (%) = (mean number of living cells in experimental group/mean number of living cells in control group) × 100%

To determine the effects of *T. gondii* on proliferation of breast cancer cells, MCF-7 cells and MDA-MB-231 cells treated with various concentration of *T. gondii* and their controls underwent growth curve analysis. After treatment of cells with *T. gondii* RH strain, total cell count in experimental groups was significantly less than that of controls at all time-points (all *P* < 0.05; Fig. 1A, E). For the breast cancer cells treated with *T. gondii* ME49 strain, the total number of cells was significantly less than that of controls until the second day after infection (Fig. 1C, G). On the second or third day after infection, the cell viability in the infected groups began to decline significantly (Fig. 1B, D, F, H). At the same time, as the concentration of *T. gondii* increased, the cell viability decreased more significantly (Fig. 1B, D, F, H).

**Fig. 2:**
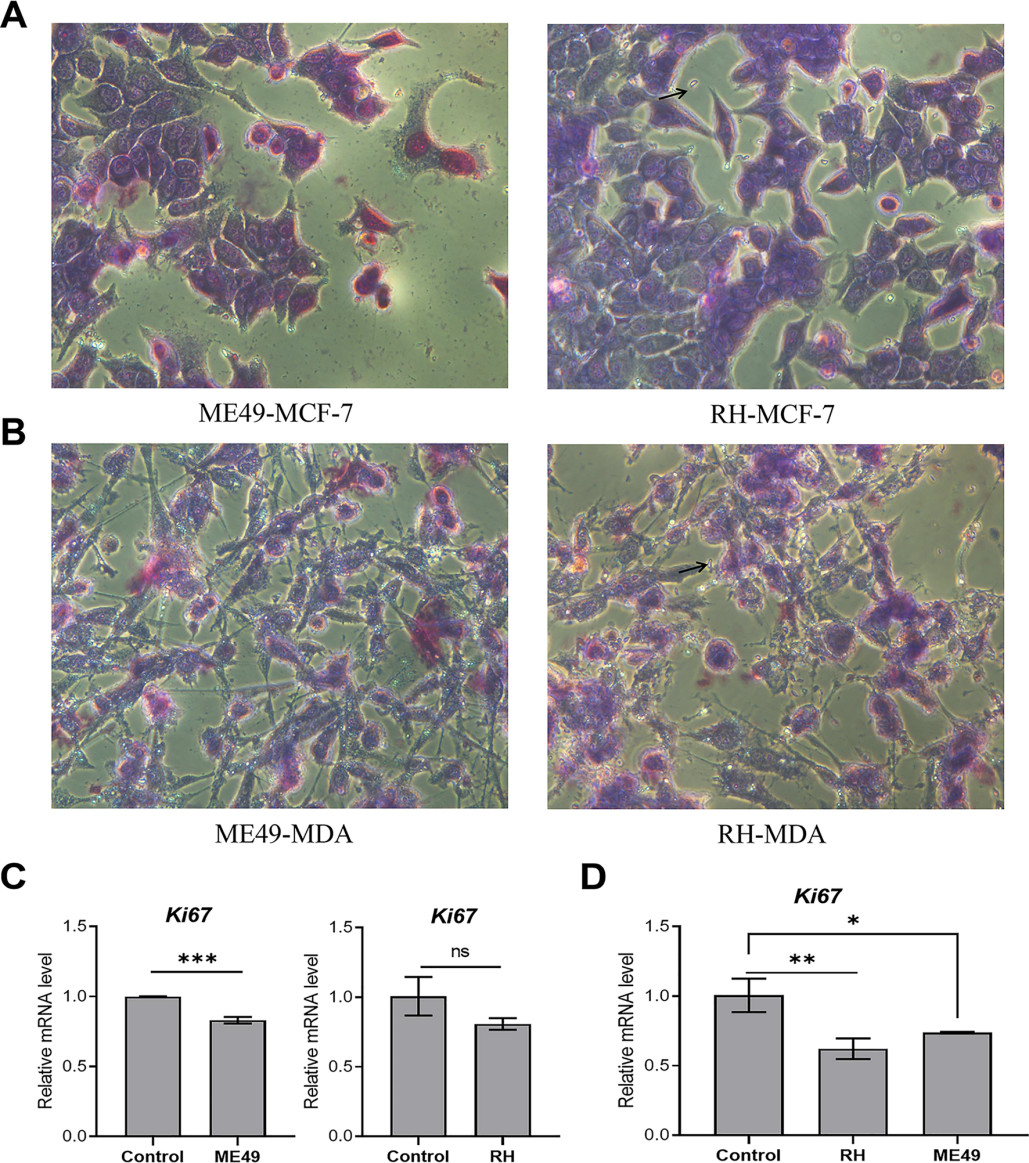
Treatment with *T. gondii* reduces the proliferation in breast cancer cells. **A** The cell morphology of MCF-7cells after 48 h of infection with *T. gondii* at 400 magnification. ME49-MCF7: MCF-7 cell infected with *T. gondii* ME49 strain (MOI = 16); RH-MCF7: MCF-7 cell infected with *T. gondii* RH strain (MOI = 4). Arrows indicate extracellular tachyzoites. **B** The cell morphology of MDA-MB-231 cells after 48 h of infection with *T. gondii* at 400 magnification. ME49-MDA: MDA-MB-231 cell infected with *T. gondii* ME49 strain (MOI = 16); RH-MDA: MDA-MB-231 cell infected with *T. gondii* RH strain (MOI = 4). Arrows indicate extracellular tachyzoites. **C** Bar graphs showing the decreased mRNA levels of *Ki67* in MCF-7 cells treated with *T. gondii* compared to controls, as determined by qRT-PCR. **D** Bar graphs showing the decreased mRNA levels of *Ki67* in MDA-MB-231 cells treated with *T. gondii* compared to controls, as determined by qRT-PCR. Data are presented as mean ± SD. ns: non-significant; *: *P* < 0.05; **: *P* < 0.01; ***: *P* < 0.001

As shown in Fig. 2A and 2B, two days after infection, breast cancer cells infected with *T. gondii* ME49 strain (MOI = 16) were not tightly arranged on the dish, and became round. In addition, the nucleus was swollen and loose. After infection with RH strain (MOI = 4), a large number of tachyzoites can be seen escaping from the cells under the microscope (Fig. 2A, B). To further confirm the suppressive effect of *T. gondii* on the proliferation, we performed qRT-PCR to detect the expression of proliferative biomarker–-*Ki67* [29]. Both *T. gondii* RH strain and ME49 strain significantly down-regulated the expression level of *Ki67* in breast cancer cells (Fig. 2C, D).

### *T. gondii* reduces the migration of breast cancer cells

**Fig. 3:**
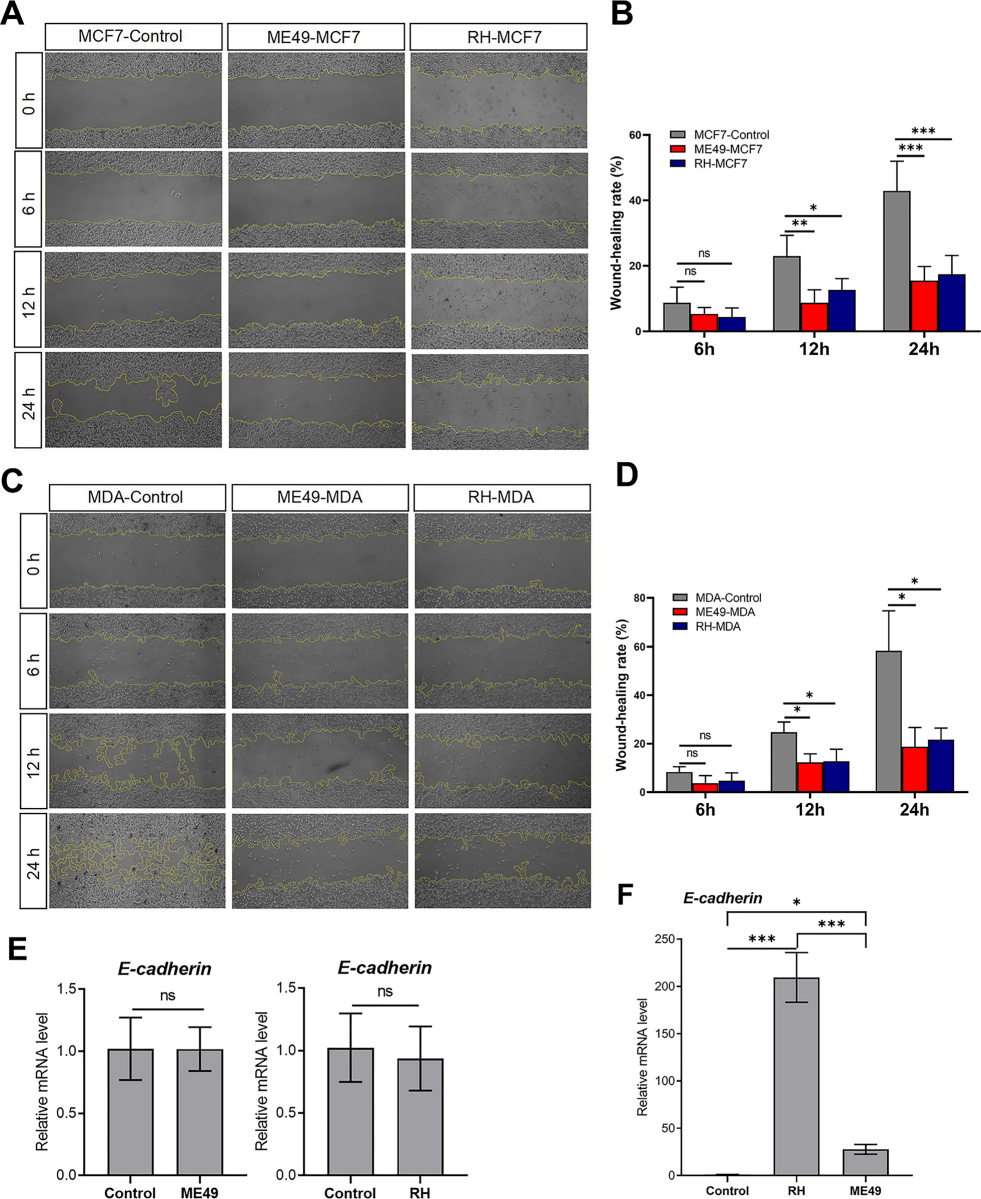
Treatment with *T. gondii* reduces the migration in breast cancer cells. **A** Cell wound healing assay showed that migration ability of MCF-7 cells was suppressed by *T. gondii*. MCF-7 cells (2 × 10^6^ cells/well) were infected with *T. gondii* ME49 strain (MOI = 16) and RH strain (MOI = 4), and the scratch changes were observed at 0, 6, 12 and 24 h. **B** Quantification of cellular migration via measurement of wound healing rate in MCF-7 cells infected with *T. gondii* as represented in A. **C** Cell wound healing assay showed that migration ability of MDA-MB-231 cells was suppressed by *T. gondii*. MDA-MB-231 cells (2 × 10^6^ cells/well) were infected with *T. gondii* ME49 strain (MOI = 16) and RH strain (MOI = 4), and the scratch changes were observed at 0, 6, 12 and 24 h. **D** Quantification of cellular migration via measurement of wound healing rate in MDA-MB-231 cells infected with *T. gondii* as represented in C. Images obtained at indicated times were representatives of three independent experiments. **E** Bar graphs showing the decreased mRNA levels of *E-cadherin* in MCF-7 cells treated with *T. gondii* compared to controls, as determined by qRT-PCR. **F** Bar graphs showing the decreased mRNA levels of *E-cadherin* in MDA-MB-231 cells treated with *T. gondii* compared to controls, as determined by qRT-PCR. Bars represent mean ± SD (n = 3). ns: non-significant; *: *P* < 0.05; **: *P* < 0.01; ***: *P* < 0.001

To test whether *T. gondii* suppresses the metastatic properties of breast cancer cells, MCF-7 cells and MDA-MB-231 cells infected with *T. gondii* and their controls underwent wound healing assays as well as expression assessment of *E-cadherin*, one of the markers of epithelial to mesenchymal transition (EMT) [30]. Wound healing assays in Fig. 3A and B showed that MCF-7 cells infected with *T. gondii* migrated significantly slower than control cells, especially 12 h after infection. Likewise, treatment with *T. gondii* caused a significant reduction in migration abilities of the MDA-MB-231 cells compared with controls (Fig. 3C, D). *T. gondii* RH strain and ME49 strain significantly up-regulated the expression level of *E-cadherin* in MDA-MB-231cells, but not in MCF-7 cells (Fig. 3E, F). Additionally, the expression level of *E-cadherin* in RH strain infected MDA-MB-231 cells was significantly higher than that in the ME49 strain infected ones (Fig. 3F).

### Dual RNA-seq on the *T. gondii* and breast cancer cells after interaction

**Fig.4:**
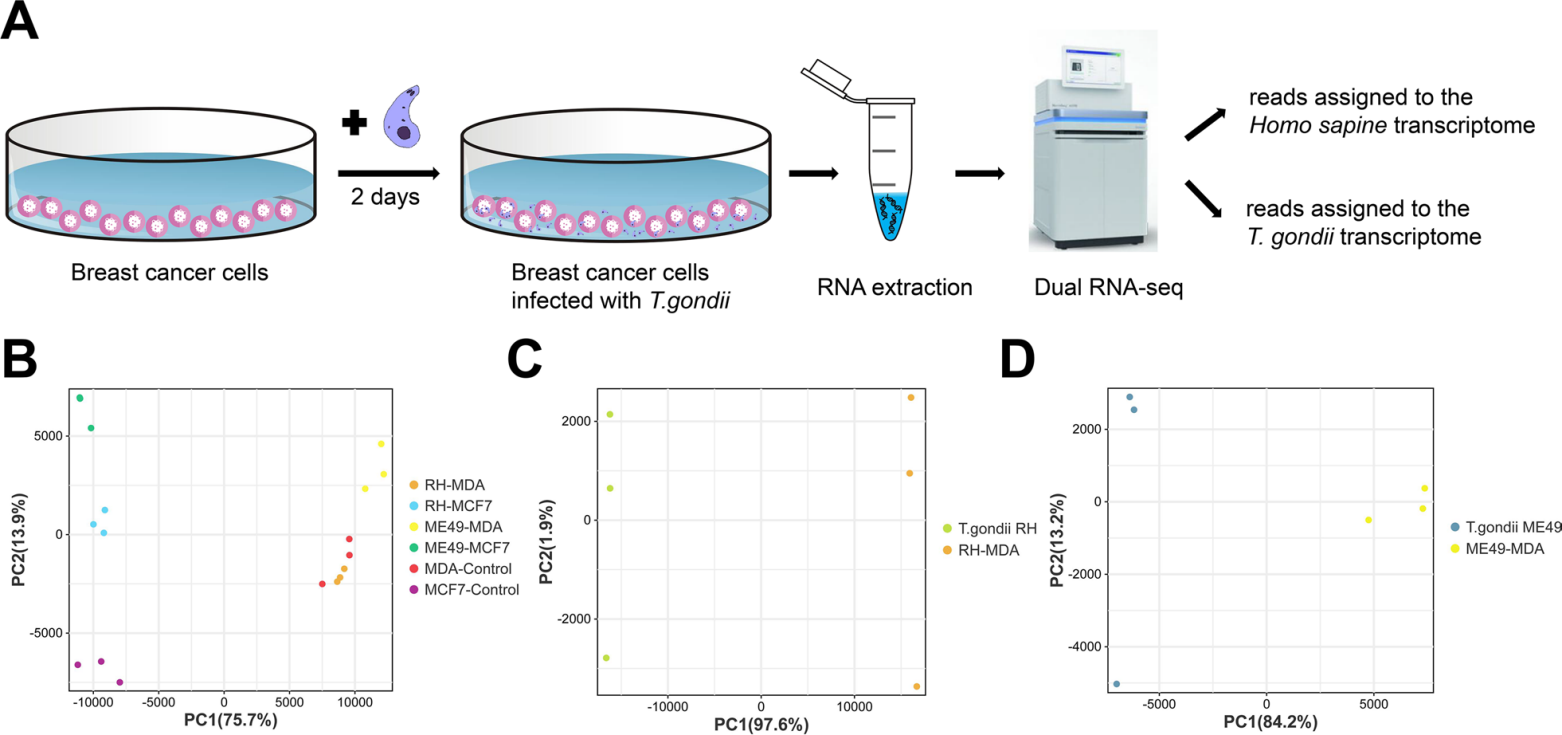
Dual RNA-seq on the *T. gondii* and breast cancer cell after their interaction. **A** Schematic of the experiment representing the main step of the infection with *T. gondii* and the time points when RNA was extracted. Libraries were created and processed through high-throughout sequencing. Reads were assigned to either the *Homo sapines* or *T. gondii* transcriptome, and DEGs were detected by DESeq2. **B** PCA of the breast cancer cells triplicate results for each group. Each replicate is represented by a circle. Each group was assigned a colour: orange (RH-MDA), light blue (RH-MCF7), yellow (ME49-MDA), dark green (ME49-MCF7), red (MDA-control), purple (MCF7-control). Note: two of the replicates of the ME49-MCF7 group were overlapped. **C** PCA of the *T. gondii* RH strain triplicate results for each group. Each replicate is represented by a circle. Each group was assigned a colour: orange (RH-MDA), light green (*T. gondii* RH). **D** PCA of the *T. gondii* ME49 strain triplicate results for each group. Each replicate is represented by a circle. Each group was assigned a colour: yellow (ME49-MDA), dark blue (*T. gondii* ME49)

To assess the transcriptome changes after the interaction between *T. gondii* and breast cancer cells, we collected triplicate RNA samples of infected breast cancer cells at 2 days post-infection (Fig. 4A). We analyzed transcriptomic profiles of the *T. gondii* and breast cancer cells. After removing low quality reads, clean reads were obtained and > 95% of the clean reads had Phred-like quality scores at the Q20 (an error probability of 0.01) and the GC-contents were about 50% (Table 1). Sequencing clean reads were mapped to the transcriptome of the *T. gondii* or human (Additional file 3: Table S2). The infected host transcriptome was compared to the non-infected host transcriptome. Reads aligned to the parasite group were compared to purified tachyzoite-derived sequencing reads. We performed a principal component analysis (PCA) to identify how each condition was clustering (Fig. 4B–D). On the host side, PCA showed that the response to same *T. gondii* strain was different for MCF-7 cells compared to MDA-MB-231 cells. At the same time, the response in the same cells was different for *T. gondii* RH strain compared to ME49 strain. On the parasite side, the PCA analysis revealed that expression of *T. gondii* was different between non-interacted and interacted group.

**Table 1:** Sequencing data quality of RNA samples

Group names	Replicates	Raw data	Clean data (%)	Q20 (%)^a^	Q30 (%)^b^	GC content (%)
MCF7-Control	1	43,008,256	42,675,908 (99.23%)	96.87	91.71	50.21
	2	42,320,664	42,053,794 (99.37%)	97.46	92.94	48.99
	3	46,664,468	46,332,242 (99.29%)	97.41	92.90	49.69
RH-MCF7	1	45,433,840	45,077,732 (99.22%)	97.22	92.47	50.72
	2	43,251,286	42,923,576 (99.24%)	97.44	92.94	52.38
	3	42,540,634	42,218,686 (99.24%)	97.30	92.66	51.47
ME49-MCF7	1	39,850,378	39,541,180 (99.22%)	97.05	92.13	51.52
	2	35,919,448	35,630,834 (99.20%)	96.93	91.88	51.47
	3	48,489,656	48,135,114 (99.27%)	97.39	92.85	50.89
MDA-Control	1	48,655,104	48,295,604 (99.26%)	96.89	90.91	50.26
	2	54,805,114	54,435,262 (99.33%)	97.70	93.41	50.65
	3	45,504,072	45,194,564 (99.32%)	96.81	91.07	50.25
RH-MDA	1	42,000,072	41,713,592 (99.32%)	96.97	91.03	48.96
	2	43,396,684	42,999,618 (99.09%)	96.50	90.50	49.50
	3	48,019,976	47,655,084 (99.24%)	96.47	90.14	49.21
ME49-MDA	1	57,835,620	57,477,664 (99.38%)	97.24	91.73	51.04
	2	43,552,936	43,212,422 (99.22%)	97.54	93.13	52.15
	3	51,301,478	50,900,190 (99.22%)	96.49	90.20	51.92
*T. gondii* RH	1	43,693,644	43,372,154 (99.26%)	97.21	92.55	53.91
	2	44,141,046	43,825,502 (99.29%)	97.40	92.96	53.75
	3	46,802,056	46,451,454 (99.25%)	97.09	92.29	53.86
*T. gondii* ME49	1	42,966,202	42,647,498 (99.26%)	97.24	92.60	53.60
	2	38,737,176	38,441,932 (99.24%)	97.23	92.51	51.70
	3	46,381,542	46,050,574 (99.29%)	97.56	93.31	53.81

^a^ Percentage is the proportion of nucleotides with a quality value > 20 in reads

^b^ Percentage is the proportion of nucleotides with a quality value > 30 in reads

**Fig. 5:**
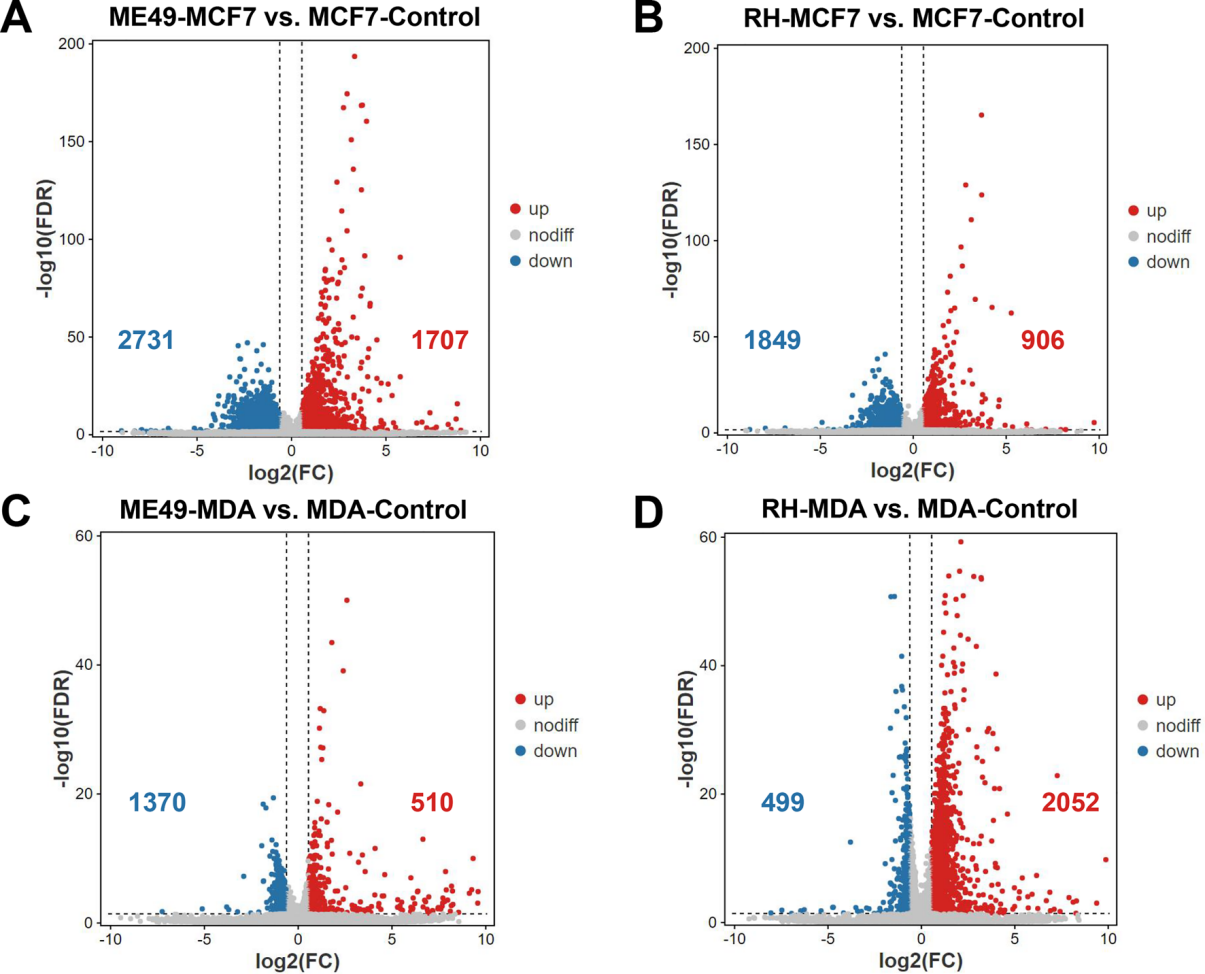
Volcano plot of transcriptome expression differences in breast cancer cells infected with *Toxoplasma gondii*. **A** MCF-7 cells infected with *T. gondii* ME49 strain. **B** MCF-7 cells infected with *T. gondii* RH strain. **C** MDA-MB-231 cells infected with *T. gondii* ME49 strain. **D** MDA-MB-231 cells infected with *T. gondii* RH strain. The filter criteria was set as fold changes ≥ 1.5 and adjusted p-value < 0.05. Red dots indicate up-regulated genes and blue dots down-regulated genes

For differential gene expression analysis, pair wise comparisons of datasets from infected breast cancer cells vs. controls were performed. As shown in volcano plot (Fig. 5A), 1707 genes were up-regulated, while 2731 genes were down-regulated in *T. gondii* ME49-infected MCF-7 cells, compared with MCF-7 controls. One thousand eight hundred and forty-nine genes were found down-regulated, while 906 genes were up-regulated in *T. gondii* RH-infected MCF-7 cells, compared with MCF-7 controls (Fig. 5B). One thousand three hundred and seventy genes were found down-regulated, while 510 genes were up-regulated in *T. gondii* ME49-infected MDA-MB-231cells, compared with MDA-MB-231 controls (Fig. 5C). Four hundred and ninety nine genes were down-regulated, while two thousand and fifty two genes were up-regulated in *T. gondii* RH-infected MDA-MB-231cells, compared with MDA-MB-231 controls (Fig. 5D).

### Functional annotation and KEGG pathway enrichment analysis of DEGs

**Fig. 6:**
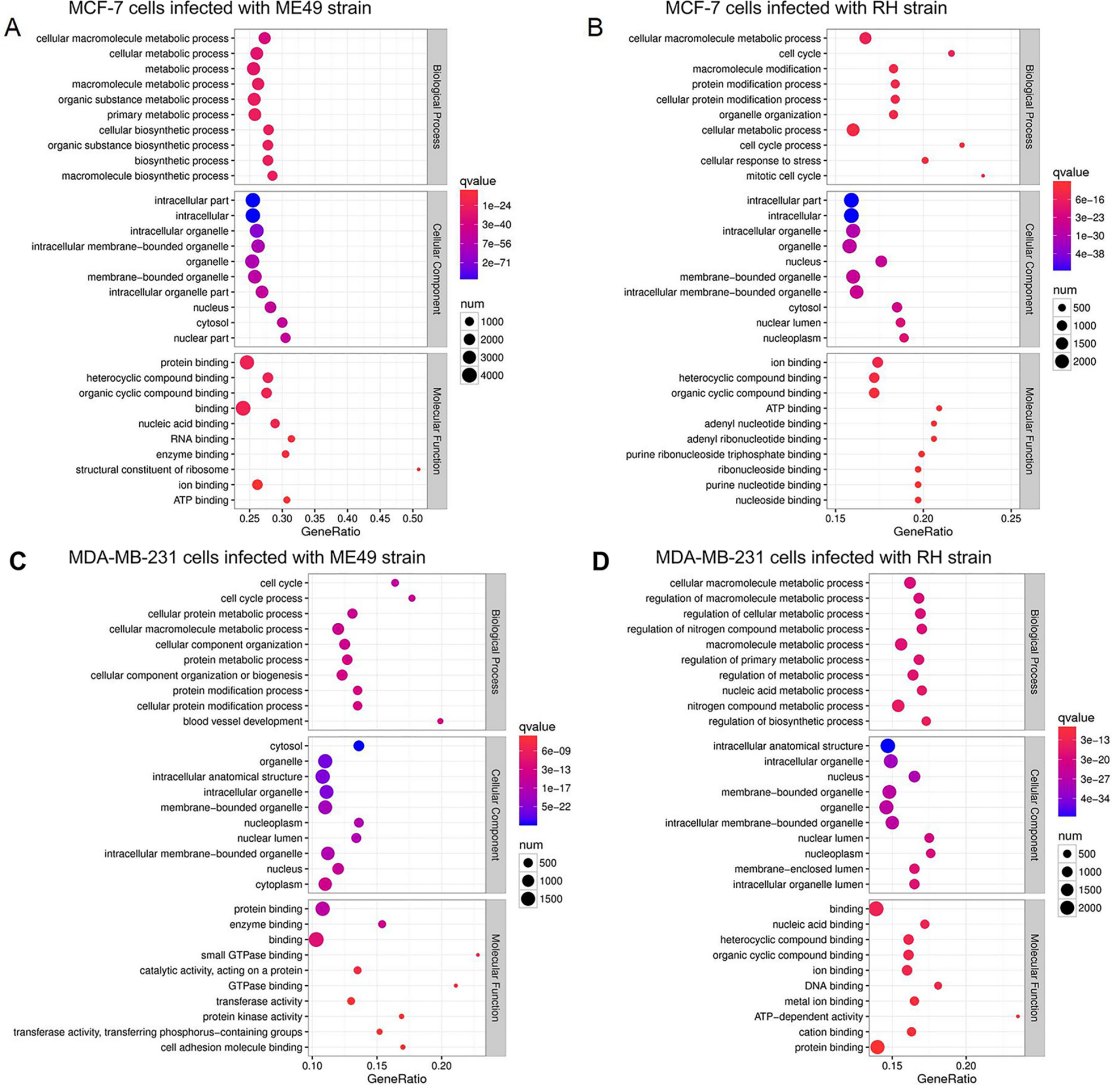
GO analysis of DEGs in breast cancer cells and *T. gondii* infected breast cancer cells. **A** MCF-7 cells infected with *T. gondii* ME49 strain. **B** MCF-7 cells infected with *T. gondii* RH strain. **C** MDA-MB-231 cells infected with *T. gondii* ME49 strain. **D** MDA-MB-231 cells infected with *T. gondii* RH strain

To better understand the roles of the identified DEGs in human breast cancer cells response to *T. gondii*, the GO and KEGG enrichment analyses were performed to identify the biological functions of the EDGs. GO analysis consists of three different aspects, namely biological process, cellular component and molecular function. Prediction terms with *q*-value (corrected *P*-value) less than 0.05 were selected and ranked by *q*-value, and the top 10 generally affected GO terms in each category are listed. Irrespective of the *T. gondii* strain used for infection, the most enriched biological process terms were related to “cellular macromolecule metabolic process” and “cell cycle” in MCF-7 cells and MDA-MB-231 cells (Fig. 6). The most enriched cell component terms were mostly about “intracellular part” in MCF-7 cells (Fig. 6A and B), while in MDA-MB-231 cells were “cytosol” and “intracellular anatomical structure” (Fig. 6C and D). As for molecular function, the most enriched terms were related to “protein binding” and “ion binding” in MCF-7 cells (Fig. 6A and B), while in MDA-MB-231 cells were “protein binding” and “binding” (Fig. 6C and D).

**Fig. 7:**
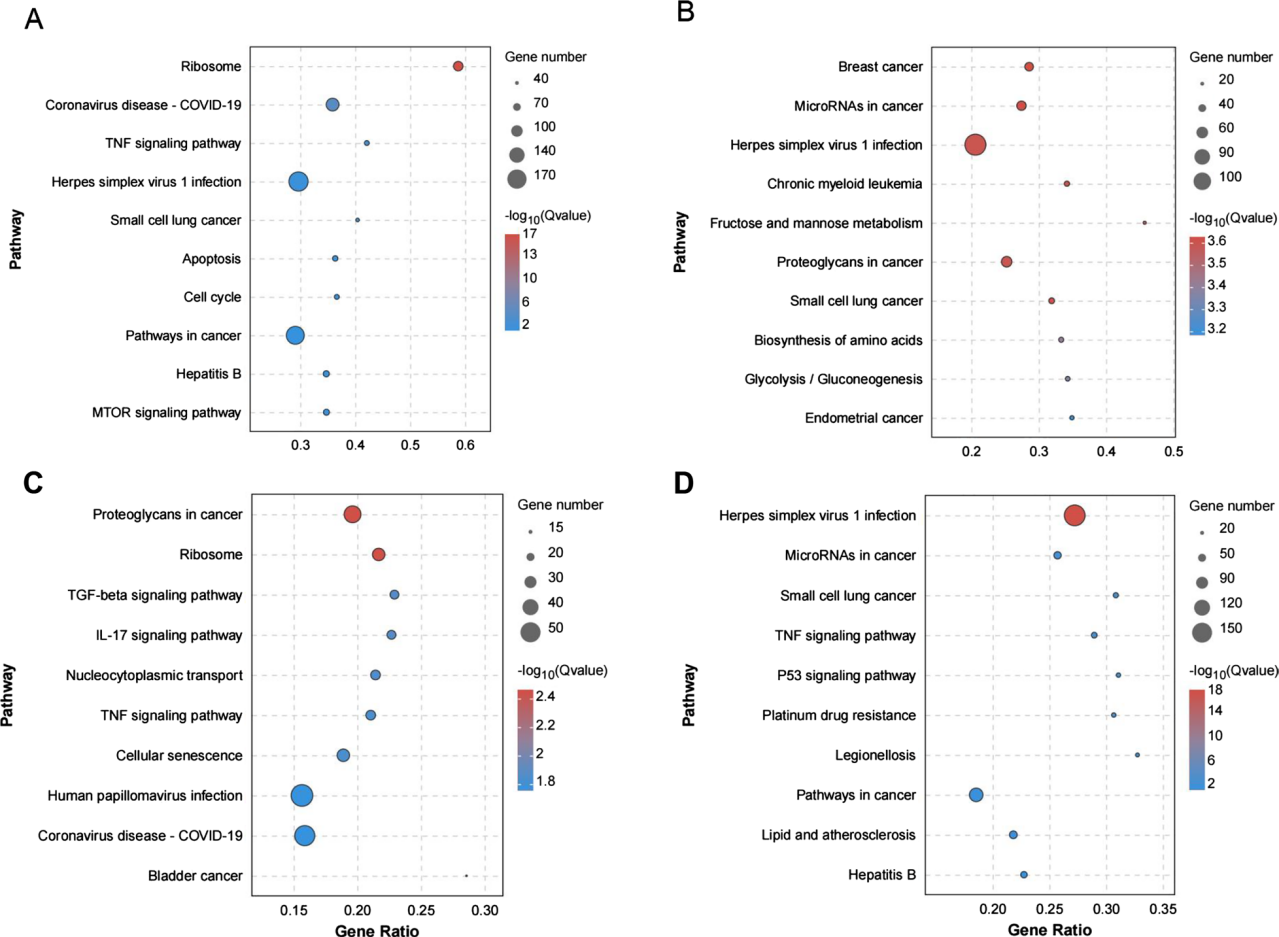
KEGG analysis of DEGs in breast cancer cells and *T. gondii* infected breast cancer cells. **A** MCF-7 cells infected with *T. gondii* ME49 strain. **B** MCF-7 cells infected with *T. gondii* RH strain. **C** MDA-MB-231 cells infected with *T. gondii* ME49 strain. **D** MDA-MB-231 cells infected with *T. gondii* RH strain

DEGs were also mapped to the terms in the KEGG database to identify signaling pathways operating during the interaction between *T. gondii* and breast cancer cells. The top 10 highly enriched pathways in breast cancer cells infected with *T. gondii* were shown in Fig. 7. After infecting by ME49 strain, “Ribosome” pathway was the most significantly regulated pathway in MCF-7 cells (Fig. 7A). For the infection by RH strain, “Breast cancer” pathway was the most significantly regulated one in MCF-7 cells (Fig. 7B). After interacting with ME49 strain, the “proteoglycans in cancer” pathway in MDA-MB-231 cells was the most significant one (Fig. 7C). For the interaction between RH strain and MDA-MB-231 cells, “Herpes simplex virus 1 infection” was the most significant pathway (Fig. 7D). In addition, the significant pathways, including pathways in cancer, cytokine-related pathways, cell cycle related pathways, were well-known contributors to cancer development.

### Identification of the hub genes in the interaction between T. gondii and breast cancer cells

**Fig. 8:**
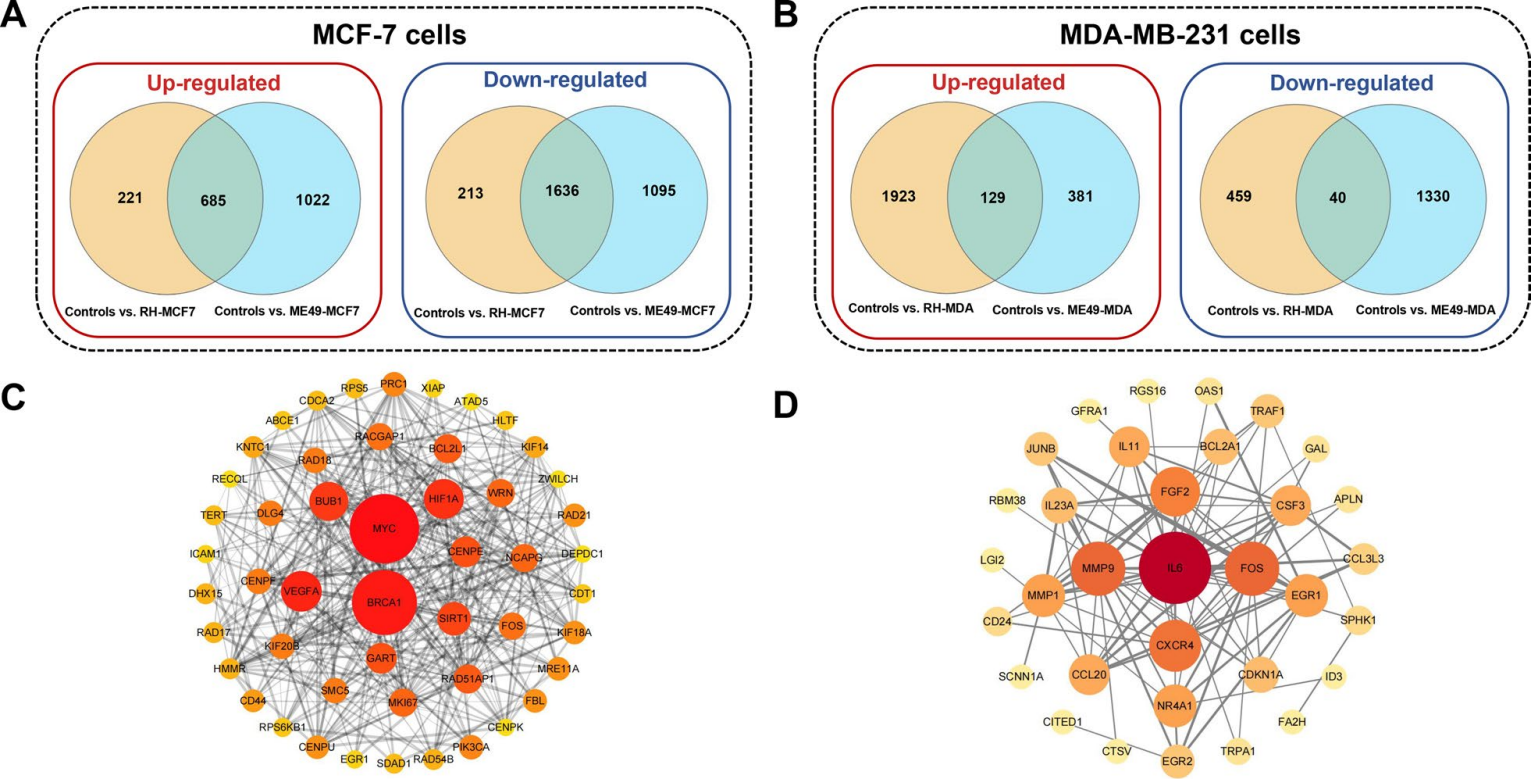
Validation of the target genes in a breast cancer cohort. **A** In the post-infected MCF-7 cells, 685 genes and 1636 genes were separately up-regulated and down-regulated. **B** In the post-infected MDA-MB-231 cells 129 genes and 40 genes were separately up-regulated and down-regulated. **C** Interaction networks of the top 50 hub genes in MCF-7 cells regulated by *T. gondii*. **D** Interaction networks of the hub genes in MDA-MB-231 cells regulated by *T. gondii*

In order to screen out the target genes regulated by *T. gondii* in breast cancer cells, the genes co-upregulated or co-downregulated by two strains were firstly identified using Venn diagrams. In total, 685 genes and 1636 genes were separately up-regulated and down-regulated in MCF-7 cells which infected with *T. gondii* (Fig. 8A). While in MDA-MB-231 cells infected with *T. gondii*, 129 genes and 40 genes were up-regulated and down-regulated, respectively (Fig. 8B). Then, we further validated these above genes in a breast cancer cohort (Table 2). In post-infected MCF-7 cells, genes out of 698 identified target genes were associated with tumor progression in the breast cancer population, including 305 up-regulated and 393 down-regulated genes. In post-infected MDA-MB-231 cells, genes out of 67 identified target genes were associated with tumor progression in the breast cancer population, including 58 up-regulated and 9 down-regulated genes. Then, the hub genes were identified based on the degree of connectivity with the cytoHubba plug-in of Cytoscape software (Fig. 8C, D). *BRCA1, MYC* and *IL-6* were identified as the top three hub genes in infected-breast cancer cells based on the connectivity of PPI analysis. We further verify the RNA-seq results using qRT-PCR to detect the expression of the hub genes (Additional file 1: Figure S1 and Additional file 2: Figure S2). Among the hub genes in post-infected MCF-7 cells identified by RNA-seq, 16 genes were confirmed by qRT-PCR and validated by KM Plotter database (Table 3). For the hub genes in post-infected MDA-MB-231 cells identified by RNA-seq, 7 genes were confirmed by qRT-PCR and validated by KM Plotter database (Table 4). Early growth response 1 (*EGR1*) was significantly up-regulated in MCF-7 cells and MDA-MB-231 cells after *T. gondii* infection irrespective of strain (Tables 3 and 4).

**Table 2:** The associations between target genes and prognosis of breast cancer were validated in the KM plotter database

	Total	Validated	Non-validated	Not included
Gene expression in the MCF-7 cells after infection				
Up-regulated	685	305	377	3
Down-regulated	1636	393	1242	1
Total	2321	698	1619	4
Gene expression in the MDA-MB-231 cells after infection				
Up-regulated	129	58	71	0
Down-regulated	40	9	31	0
Total	169	67	102	0

**Table 3:** The hub genes regulated by *T. gondii* in MCF-7 cells were validated by qRT-PCR

Genes	Hazard ratio (95% CI)^a^	qRT-PCR (relative expression)			RNA-seq (FPKM)		
		Controls	ME49-infected	RH-infected	Controls	ME49-infected	RH-infected
Genes that are up-regulated by *T. gondii*							
*MYC*	0.59 (0.47, 0.74)	1.01	3.59	3.24	37.14	119.43	85.44
*RPS5*	0.69 (0.55, 0.87)	1.00	1.18	1.34	656.63	1171.19	990.45
*ICAM1*	0.70 (0.55, 0.89)	1.03	63.84	5.45	1.21	56.06	4.08
*EGR1*	0.59 (0.47, 0.74)	1.01	7.07	8.08	9.66	33.78	39.39
*SOD2*	0.77 (0.60, 0.98)	1.02	10.03	2.25	28.55	163.03	48.52
*BNIP3*	0.70 (0.56, 0.89)	1.02	3.23	5.20	24.26	76.55	101.14
*RPS12*	0.72 (0.57, 0.90)	1.01	1.67	1.73	424.46	842.89	649.95
*AHCY*	0.54 (0.43, 0.67)	1.01	1.48	1.86	116.95	219.62	212.79
*JUNB*	0.73 (0.58, 0.91)	1.02	2.27	3.02	74.25	173.24	133.80
*NME2*	0.53 (0.42, 0.66)	1.02	1.45	1.70	1093.00	2030.18	1671.80
Genes that are down-regulated by *T. gondii*							
*KI67*	1.49 (1.19, 1.87)	1.00	0.83	0.81	31.75	11.23	15.02
*RACGAP1*	1.60 (1.26, 2.02)	1.04	0.49	0.50	92.84	35.89	44.23
*PRC1*	1.71 (1.37, 2.14)	1.01	0.64	0.70	108.63	46.79	55.05
*RAD21*	1.35 (1.07, 1.70)	1.01	0.90	0.99	134.41	50.67	73.46
*DHX15*	1.58 (1.26, 2.00)	1.03	1.02	0.98	63.73	33.28	41.67
*RPS6KB1*	1.55 (1.24, 1.94)	1.04	0.81	1.04	201.17	69.72	122.90

^a^ These results were from the KM plotter database. The low expression group was used as the reference group

**Table 4:** The hub genes regulated by *T. gondii* in MDA-MB-231 cells were validated by qRT-PCR

Genes	Hazard ratio (95% CI)^a^	qRT-PCR (relative expression)			RNA-seq (FPKM)		
		Controls	ME49-infected	RH-infected	Controls	ME49-infected	RH-infected
Genes that are up regulated by* T. gondii*							
*IL6*	0.79 (0.63, 0.99)	1.01	18.88	5.58	90.33	138.15	217.73
*EGR1*	0.59 (0.47, 0.74)	1.05	162.30	66.92	1.64	10.19	40.00
*CDKN1A*	0.58 (0.46, 0.73)	1.02	7.26	3.17	97.48	229.97	211.58
*JUNB*	0.73 (0.58, 0.91)	1.13	13.63	9.39	24.83	47.98	58.85
*CCL3L1*	0.72 (0.56, 0.93)	1.06	123.60	114.80	29.99	73.42	140.15
*ID3*	0.71 (0.56, 0.90)	1.03	0.99	2.13	10.73	34.02	24.27
G enes that are down regulated by *T. gondii*							
*PRC1*	1.71 (1.37, 2.14)	1.02	0.24	0.84	83.91	48.207	54.31

^a^ These results were from the KM plotter database. The low expression group was used as the reference group

To further identify which proteins secreted by *T. gondii* were involved in the interaction with breast cancer cells, we used the PHI database to annotate genes which were dysregulated significantly during the interaction. As shown in Table 5, after interacting with breast cancer cells, *T. gondii* RH strain increased the secretion of rhoptry proteins 16 (ROP16) and ROP18, while decreasing the secretion of Calreticulin (crt) and Toxoplasma inhibitor of STAT1-dependent transcription (TgIST). For *T. gondii* ME49 strain, it decreased the secretion of *TgIST*, dense granule antigen 15 (GRA15), GRA24 and Micronemal protein 13 (MIC13).

**Table 5:** Transcriptome changes of *T. gondii* after interaction with breast cancer cells annotation results of PHI database ^a^

Transcriptome	After interaction with MCF-7 cells		After interaction with MDA-MB-231 cells	
	*T. gondii* RH	*T. gondii* ME49	*T. gondii* RH	*T. gondii* ME49
Gene counts				
Up-regulated	1369	843	1422	902
Down-regulated	1648	888	1569	1341
PHI database ^b^ annotation results genes which interacted with Human genes				
Up-regulated	*ROP16* *ROP18*	-	*ROP16* *ROP18*	-
Down-regulated	*crt*	*TgIST*	*crt*	*TgIST, GRA15, GRA24, MIC13*

^a^ The filter criteria was set as fold changes ≥ 1.5 and adjusted *p*-value < 0.05;

^b^ PHI, Pathogen Host Interaction

## Discussion

Previous studies using attenuated or wild *T. gondii* strain in the treatment of solid tumors, have produced certain therapeutic effects against advanced metastatic cancers, such as ovarian cancer and pancreatic cancer. Recently, our previous study found that anti-*T. gondii* IgG could improve the survival of breast cancer patients [9], but the underlying mechanism was still unclear. In this study, we firstly utilized dual RNA-seq to analyze the transcriptome expression in the interaction between breast cancer cells and *T. gondii*.

Our results showed that *T. gondii* exerts a time-dependent or a concentration-dependent inhibitory effect on the proliferation of breast cancer cells. These results are consistent with previous studies. For example, Peng et al. found that *T. gondii* RH strain not only significantly inhibited the growth of MCF-7 cells after 48 h treating, but also induced the apoptosis of MCF-7 cells [31]. In our study, Ki-67 levels were decreased significantly in MDA-MB-231 cells infected with RH strain, but not in MCF-7 cells. This result may be due to differences in the ability of the two types of cells to proliferate [32]. According to a previous research, the expression of Ki-67 in MDA-MB-231 was 100%, while the expression of Ki-67 in MCF-7 cell was only 90% [32]. Therefore, the expression of Ki-67 might be suppressed more apparently in MDA-MB-231 cells than that in MCF-7 cells. In addition to inhibiting the growth of breast cancer cells, *T. gondii* can also significantly inhibit other tumor cells such as prostate cancer cells, esophageal cancer cells, and lung cancer cells [33]. The underlying mechanisms may be that *T. gondii* tachyzoites can induced the G2/M arrest of cancer cells by cyclinB1 [34].

In addition, *T. gondii* can significantly inhibited the migration of breast cancer cells. E-cadherin, as a key protein in cellular adhesion, its down-regulation is linked to cancer progression [35]. The expression of E-cadherin was significantly different in different breast cancer cell lines [36]. For example, all breast cancer cell lines except MDA-MB-231 expressed robust levels of E-cadherin [36]. Because of *T. gondii’* s limited ability to inhibit metastasis, the inhibitory effect on cells with high expression of E-cadherin is limited, while the effect might be apparent in cells which showed low expression of E-cadherin. Moreover, *T. gondii* RH strain is a highly virulent strain, while ME49 strain is an avirulent strain, so RH strain might inhibit the migration of breast cancer cells more obviously than ME49 strain. Previous studies have suggested that this inhibiting effect may be closely related to *T. gondii* lysate antigens or secreted proteins [37], such as Toxoplasma profilin (TgPLP) [38], rhoptry proteins (ROPs) [39], and dense granule antigens (GRAs) [39, 40]. For example, TgPLP can increase the level of antigen-presenting cell markers in bone marrow-derived macrophages by activating the MyD88 pathway, resulting in increasing the production of IL-12 and promoting their phagocytosis of tumor cells [38].

Previous transcriptome sequencing results revealed that *T. gondii* significantly altered the expression of transcriptome of host. Both acute and chronic infection with *T. gondii* lead to the dysregulation of multiple metabolic pathways [41, 42], and eventually activated the certain immune response signaling pathways [43]. In our study, we also found that the transcriptomic expression of breast cancer was significantly changed after interaction with *T. gondii*. Particularly, the number of up-regulated and down-regulated genes expressed in MCF-7 cells was significantly higher than that in MDA-MB-231 cells after infection with *T. gondii*, which may be due to the heterogeneity of breast cancer cells [32]. In the present study, KEGG analysis showed the certain signaling pathways were regulated as follow: ribosome, breast cancer pathway, IL-17 signaling pathway, and coronavirus disease (COVID-19) pathway. Particularly, ribosome signaling pathway was also considered as one of the key signaling pathway by GSEA in our study. Ribosome signaling pathway is closely related to cell cycle, especially in G1 phase, a large number of ribosomes are generated, and the generation of ribosomes can promote the progress of cell cycle [44]. Therefore, when the ribosome signaling pathway is dysregulated by *T. gondii*, the cell cycle will be halted, and the normal proliferation of breast cancer cells will be inhibited [34].In addition, in consistent with the previous studies, our study also found that the breast cancer signaling pathway was significantly dysregulated after interaction with *T. gondii* [13].

Our previous study has found that interleukin-17 (IL-17) was one of the main contributors to the interaction between *T. gondii* and breast cancer prognosis [9]. Likewise, numerous studied have shown that IL-17 plays an important role in promoting tumor proliferation, invasion and metastasis, which is closely related to poor prognosis [45]. The effects of IL-17 on breast cancer can be divided into direct and indirect effects: IL17 could directly change the gene-expression profile and the behavior of nonmetastatic tumor cells, causing tumor growth in vivo [46]; IL-17 may also promote tumor progression by recruiting neutrophils to tumor tissue. Neutrophils secrete a variety of proteins that degrade the extracellular matrix, making it easier for tumor cells to invade other sites [47].

To explore the hub genes in breast cancer cells during the interaction, PPI analysis were further performed. *BRCA1, MYC* and *IL-6* were identified as the top three hub genes based on the connectivity. For the gene *BRCA1*, it is considered not only playing a vital role on the breast cancer pathway [48], but also involving in the ribosome biogenesis [49]. Activation of *MYC* [50] and *IL-6* [51] has been widely reported in breast cancer progression. However, in our study, how these key genes regulate the signaling pathways still needs further study. Unlike these three top three genes, Early growth response 1 (*EGR1*) was further confirmed by qRT-PCR and validated by KM Plotter database in this study. *EGR1* was significantly up-regulated in MCF-7 cells and MDA-MB-231 cells after *T. gondii* infection irrespective of strain*.* The *EGR1* gene encodes a protein belonging to the early growth response (EGR) protein family, a family of zinc finger transcription factors, which can directly regulate several tumor suppressors such as transforming growth factor beta 1 (*TGFβ1*), tumor protein p53 (*p53*), and phosphatase and tensin homolog, *PTEN*) [52]. Survival analysis revealed upregulated EGR1 was remarkably associated with favorable relapse-free survival (RFS) among breast cancer patients [53].

During the interaction of *T. gondii* and breast cancer cells, not only the transcriptomic expression of breast cancer cells was significantly changed, but also the transcriptome of *T. gondii* was significantly changed. After dual RNA-seq and annotated by the PHI-base, we found that the expression of *ROP16* and *ROP18* in *T. gondii* increased, while the expression of *crt*, *TgIST*, *GRA15*, *GRA24* and *MIC13* decreased.

ROP16 can polyubiquitinate STING, resulting in inactivate cGAS-STING signaling pathway [54]. In this way, STING was inactivated and subsequently decreased the secretion of inflammatory cytokines, downregulating the STAT1 and NF-κB pathways in brain metastatic cells, thereby suppressing the brain metastasis of breast cancer and lung cancer [55, 56].

The secretion of ROP18 and dense granule 24 (GRA24) proteins by *T. gondii* activates antitumor immunity through the IL-12/interferon-gamma (IFN-γ) TH1 axis, as well as CD4 + and CD8 + T cells [39]. However, GRA15 proteins secreted by *T. gondii* activated the NF-κB signaling, inducing the secretion of interleukin-6 (IL-6) from immune cells [57]. IL-6 played pivotal roles in the inflammation, causing the chronic inflammation, which promotes the progression of tumor [51]. In the present study, we found that the secretion of GRA15 by *T. gondii* reduced significantly after interaction with breast cancer cells. Therefore, *T. gondii* might suppress the proliferation and migration of breast cancer cells through reducing the secretion of GRA15.

Nevertheless, the present study has some limitations. Firstly, the experiments on the effect of *T. gondii* on breast cancer were in vitro experiments, not in vivo experiments. However, the results of the in vitro experiments in this study were consistent with the results of the in vivo experiments in most previous studies [37]. Further researches would be needed to explore the exact mechanism of *T. gondii* suppressing the progression of breast cancer using animal models or human tissue. Secondly, we conducted our experiments only using Luminal A (MCF-7 cells) and triple negative (MDA-MB-231 cells) breast cancer cells, which may not be fully representative of all breast cancer types. Considering the heterogeneity of breast cancer, it is necessary to further explore the association between *T. gondii* and breast cancer in multiple breast cancer cells or tissues.

## Conclusions

This study suggested that *T. gondii* was able to inhibit the growth and migration of breast cancer cells by transcriptionally regulating several signaling pathways which were related to the growth and metabolism such as ribosome and the IL-17 signaling pathway. During the interaction between *T. gondii* and breast cancer, the expression of transcriptome of *T. gondii* was significantly changed, secreting several proteins involving into the inhibiting effect on breast cancer cells. The findings of these mechanisms will help *T. gondii* to be used as a potential treatment to improve the prognosis of breast cancer patients.

### Supplementary Information

**Additional file 1.** Figure S1.

**Additional file 2.** Figure S2.

**Additional file 3: Table S1.** Gene name and primers used in qRT-PCR. **Table S2.** Results of alignment of sequencing data with the reference genome.

## Data Availability

The raw sequence data reported in this study have been deposited in the Genome Sequence Archive in National Genomics Data Center (https://ngdc.cncb.ac.cn), China National Center for Bioinformation / Beijing Institute of Genomics, Chinese Academy of Sciences, under the accession number: HRA004430 and CRA010759. Any other raw data used and analyzed in this study were available from the corresponding author on request.

## Abbreviations

*T. g**ondii*: *Toxoplasma gondii*
DEGs: Differentially expressed genes
GO: Gene Ontology
KEGG: Kyoto Encyclopedia of Genes and Genomes
GSEA: Gene Set Enrichment Analysis
PPI: Protein–Protein Interaction Networks analysis
PHI-base: Pathogen Host Interaction database
IL-17: Interleukin-17
IL-9: Interleukin-9
IL-12: Interleukin-12
IFN- γ: Interferon-γ
HFF: Human foreskin fibroblast cell
MOI: Multiplication of infection
GEO: Gene Expression Omnibus
EGA: European genome-phenome archive
KM plotter: Kaplan–Meier plotter
ROP16: Rhoptry proteins 16
ROP18: Rhoptry proteins 18
crt: Calreticulin
TgIST: Toxoplasma inhibitor of STAT1-dependent transcription
GRA15: Dense granule antigen 15
GRA24: Dense granule antigen 24
MIC13: Micronemal protein 13
TgPLP: Toxoplasma profiling
EGR1: Early growth response 1
TGFβ1: Transforming growth factor beta 1
PTEN: Phosphatase and tensin homolog
RFS: Relapse-free survival
HR: Hazard ratio
