# Supplementary figures and images for: Toxoplasma gondii suppresses proliferation and migration of breast cancer cells by regulating their transcriptome
Source: Cancer Cell Int. 2024 Apr 23;24:144. doi: 10.1186/s12935-024-03333-1 (PMC11040860; doi:10.1186/s12935-024-03333-1)

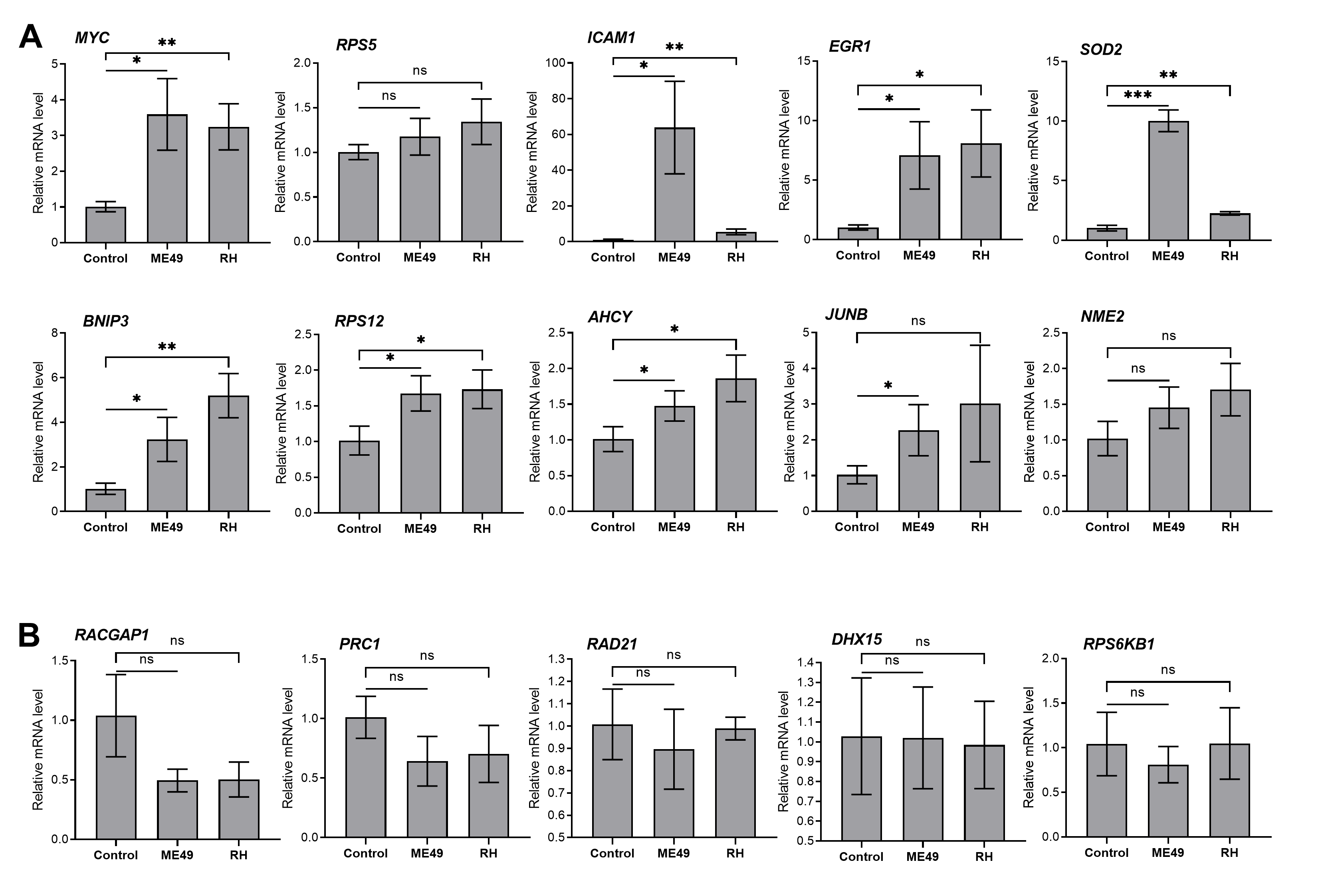

Supplement: Supplementary file 1 — Additional file 1. Figure S1. [file 12935_2024_3333_MOESM1_ESM.tif]

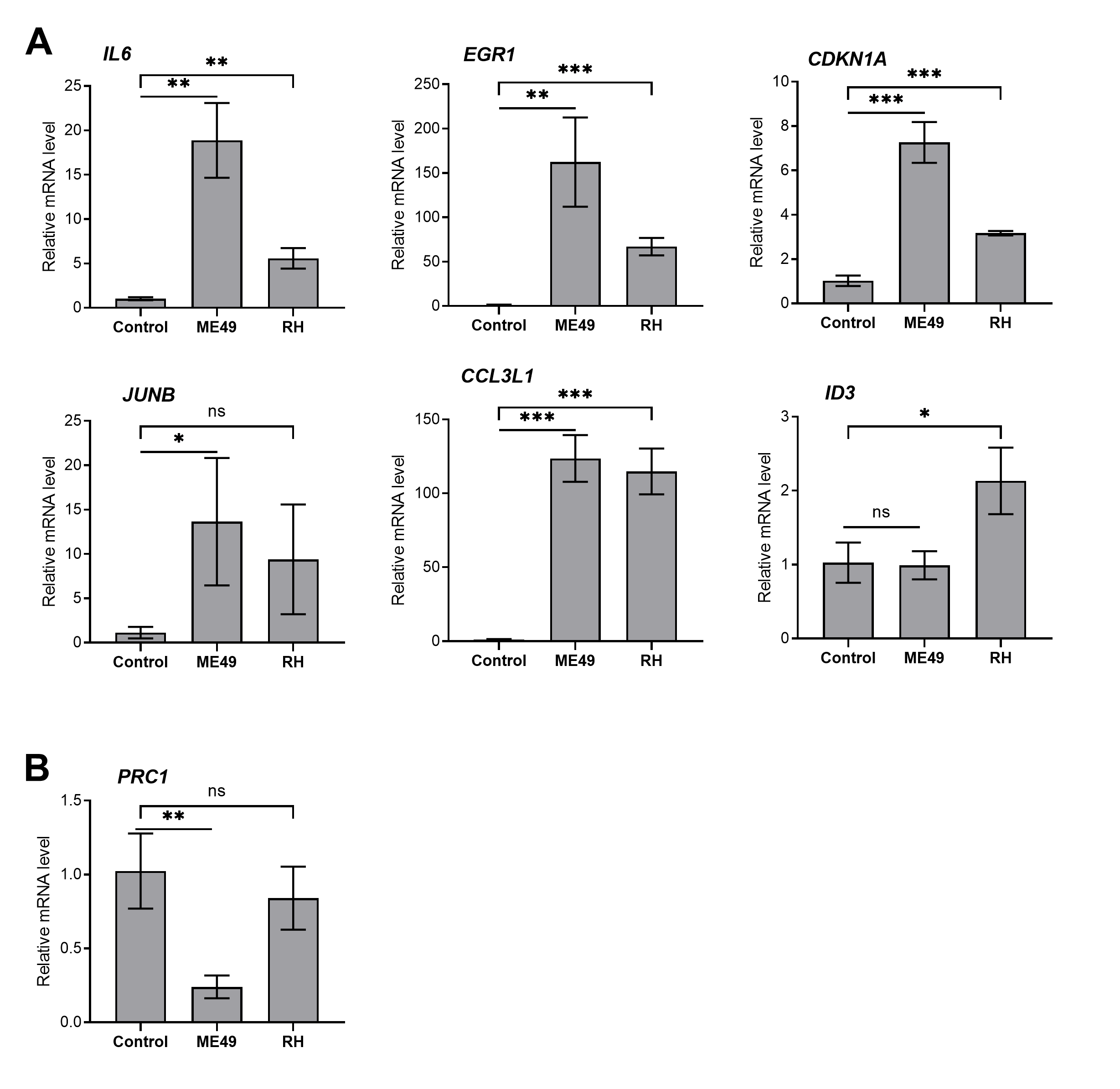

Supplement: Supplementary file 2 — Additional file 2. Figure S2. [file 12935_2024_3333_MOESM2_ESM.tif]
