# Supplementary material for: Toxoplasma gondii suppresses proliferation and migration of breast cancer cells by regulating their transcriptome
Source: Cancer Cell Int. 2024 Apr 23;24:144. doi: 10.1186/s12935-024-03333-1 (PMC11040860; doi:10.1186/s12935-024-03333-1)
Supplement: Supplementary file 3 — Additional file 3: Table S1. Gene name and primers used in qRT-PCR. Table S2. Results of alignment of sequencing data with the reference genome. [file 12935_2024_3333_MOESM3_ESM.docx]

**Table S1** Gene name and primers used in qRT-PCR

| Gene | Primer name* | Primer sequence（5' to 3'） |
| --- | --- | --- |
| Beta-actin | Beta-actin-F | CTGGAACGGTGAAGGTGACA |
|  | Beta-actin-R | AAGGGACTTCCTGTAACAACGCA |
| Ki-67 | Ki-67-F | ACGCCTGGTTACTATCAAAAGG |
|  | Ki-67-R | CAGACCCATTTACTTGTGTTGGA |
| E-cadherin | E-cadherin-F | CGAGAGCTACACGTTCACGG |
|  | E-cadherin-R | GGGTGTCGAGGGAAAAATAGG |
| MYC | MYC-F | GGCTCCTGGCAAAAGGTCA |
|  | MYC-R | CTGCGTAGTTGTGCTGATGT |
| RPS5 | RPS5-F | ATGACCGAGTGGGAGACAG |
|  | RPS5-R | GCTTTGCGGAAGCGTTTGG |
| ICAM1 | ICAM1-F | ATGCCCAGACATCTGTGTCC |
|  | ICAM1-R | GGGGTCTCTATGCCCAACAA |
| EGR1 | EGR1-F | GGTCAGTGGCCTAGTGAGC |
|  | EGR1-R | GTGCCGCTGAGTAAATGGGA |
| SOD2 | SOD2-F | GCTCCGGTTTTGGGGTATCTG |
|  | SOD2-R | GCGTTGATGTGAGGTTCCAG |
| BNIP3 | BNIP3-F | CAGGGCTCCTGGGTAGAACT |
|  | BNIP3-R | CTACTCCGTCCAGACTCATGC |
| RPS12 | RPS12-F | TGCTGGAGGTGTAATGGACG |
|  | RPS12-R | GGCGCTTGTCTAAGGCTTTG |
| AHCY | AHCY-F | ATTCCGGTGTATGCCTGGAAG |
|  | AHCY-R | GAGATGCCTCGGATGCCTG |
| JUNB | JUNB-F | ACGACTCATACACAGCTACGG |
|  | JUNB-R | GCTCGGTTTCAGGAGTTTGTAGT |
| NME2 | NME2-F | ACCTCTTATTCATAGACCCA |
|  | NME2-R | AGATTCAAAGCCAGGCACCAT |
| RACGAP1 | RACGAP1-F | ATGATGCTGAATGTGCGGAAT |
|  | RACGAP1-R | CGCCAACTGGATAAATTGGACTT |
| PRC1 | PRC1-F | ATCACCTTCGGGAAATATGGGA |
|  | PRC1-R | TCTTTCTGACAGACGGATATGCT |
| RAD21 | RAD21-F | GGATAAGAAGCTAACCAAAGCCC |
|  | RAD21-R | CTCCCAGTAAGAGATGTCCTGAT |
| DHX15 | DHX15-F | GGGGACCGATGGGAAGGAT |
|  | DHX15-R | TAGCATTTGTTGAAGCTCGCA |
| RPS6KB1 | RPS6KB1-F | CGGGACGGCTTTTACCCAG |
|  | RPS6KB1-R | TTTCTCACAATGTTCCATGCCA |
| IL6 | IL6-F | ACTCACCTCTTCAGAACGAATTG |
|  | IL6-R | CCATCTTTGGAAGGTTCAGGTTG |
| CDKN1A | CDKN1A-F | TGTCCGTCAGAACCCATGC |
|  | CDKN1A-R | AAAGTCGAAGTTCCATCGCTC |
| CCL3L1 | CCL3L1-F | CACCTCCCGACAGATTCCAC |
|  | CCL3L1-R | GGTCACTGACGTATTTCTGGAC |
| ID3 | ID3-F | GAGAGGCACTCAGCTTAGCC |
|  | ID3-R | TCCTTTTGTCGTTGGAGATGAC |

*Forward (F) and reverse (R) primers.

**Table S2** Results of alignment of sequencing data with the reference genome

| Group names | Replicates | The proportion of reads assigned to the *Homo sapines* genes (%) | The proportion of reads assigned to the *T. gondii* genes (%) | The proportion of the unmapped reads (%) |
| --- | --- | --- | --- | --- |
| MCF7-Control | 1 | 91.64 | — | 8.36 |
|  | 2 | 92.40 | — | 7.60 |
|  | 3 | 91.85 | — | 8.15 |
| RH-MCF7 | 1 | 78.93 | 14.84 | 6.23 |
|  | 2 | 76.99 | 16.91 | 6.10 |
|  | 3 | 75.71 | 17.70 | 6.59 |
| ME49-MCF7 | 1 | 76.64 | 14.43 | 8.93 |
|  | 2 | 74.44 | 17.53 | 8.03 |
|  | 3 | 72.73 | 18.82 | 8.45 |
| MDA-Control | 1 | 92.31 | — | 7.69 |
|  | 2 | 92.96 | — | 7.04 |
|  | 3 | 91.59 | — | 8.41 |
| RH-MDA | 1 | 85.12 | 6.54 | 8.34 |
|  | 2 | 84.55 | 5.90 | 9.55 |
|  | 3 | 83.89 | 6.64 | 9.46 |
| ME49-MDA | 1 | 87.04 | 4.34 | 8.62 |
|  | 2 | 88.98 | 3.48 | 7.54 |
|  | 3 | 88.07 | 2.65 | 9.29 |
| *T. gondii* RH | 1 | — | 94.69 | 5.31 |
|  | 2 | — | 94.41 | 5.59 |
|  | 3 | — | 94.57 | 5.43 |
| *T. gondii* ME49 | 1 | — | 86.72 | 13.28 |
|  | 2 | — | 44.29 | 55.71 |
|  | 3 | — | 90.19 | 9.81 |
